# Using Mixed-Methods Research to Address Stagnant Operative Mortality Rates in Congenital Heart Surgery

**DOI:** 10.1097/CCE.0000000000001435

**Published:** 2026-07-01

**Authors:** Alyssia Venna, Mitchell C. Haverty, Areen Almarkhan, Alix Fetch, Janet Kreutzer, Lisa A. Hom, David L. Wessel, Ashraf S. Harahsheh, Yuliya Domnina, Yves d’Udekem

**Affiliations:** 1 Division of Cardiovascular Surgery, Children’s National Hospital, Washington, DC.; 2 Heart Research Institute, Children’s National Hospital, Washington, DC.; 3 Division of Critical Care Medicine, Children’s National Hospital, Washington, DC.; 4 Division of Cardiology, Department of Pediatrics, Children’s National Hospital, Washington, DC.

**Keywords:** congenital heart surgery, mixed methods, operative mortality, outcomes, quality improvement

## Abstract

**IMPORTANCE:**

Operative mortality rates in the congenital heart disease population have remained stagnant at 2.8% for the last decade, prompting a mixed-methods exploration into what institutional factors might be at play.

**OBJECTIVES:**

After experiencing 0% operative mortality for 365 days, we aimed to qualitatively identify institutional factors impacting operative mortality and compare them to perceived reasons for operative mortality.

**DESIGN:**

A convergent mixed-methods design was used to compare quantitative and qualitative data.

**SETTING:**

This study was performed in a single-center pediatric heart center by a multidisciplinary cardiac research team between January 2020 and June 2024.

**PARTICIPANTS:**

A retrospective review of 43 operative mortality records was conducted to determine patient characteristics and reasons for operative mortality. Semi-structured interviews were conducted with staff.

**ANALYSIS:**

Quantitative data were analyzed using descriptive statistics to describe patient characteristics and reasons for perceived operative mortality. Qualitative data were analyzed using reflexive thematic analysis to identify themes related to institutional factors influencing operative mortality. Quantitative and qualitative data were compared by matching reasons for each operative mortality to qualitative themes in a joint display. A post hoc descriptive analysis was performed to identify additional perceived reasons for operative mortality following the 0% operative mortality period.

**RESULTS:**

Records were assigned up to five reasons for operative mortality: technical failure, communication issues, intractable disease, management strategy, and decision-making. Semi-structured interviews identified institutional factors influencing operative mortality. Reasons for operative mortality were technical failure (*n* = 19 [22%]), intractable disease (*n* = 19 [22%]), communication (*n* = 18 [21%]), management strategy (*n* = 15 [18%]), and decision-making (*n* = 14 [16%]). Three qualitative themes emerged: 1) Implementing Best Medical Practices, 2) Fostering Healthy Interpersonal Relationships, and 3) Building a Responsive Organizational Structure. Upon comparison, factors encompassed by theme 1 were most comparable when the reason for operative mortality was technical failure, intractable disease, management strategy, or decision-making); theme 2 was most comparable when the reason was communication. Theme 3 was only related to one instance of operative mortality. A post hoc analysis revealed most subsequent deaths following the 0% operative mortality period were attributed to intractable disease.

**CONCLUSIONS:**

Efforts to implement best medical practices are critical to improve operative mortality. A considerable effort should also be made to foster healthy relationships among staff. Building a responsive organizational structure may indirectly influence outcomes and should be further explored.

**KEY POINTS**

**Question**: How do the perceived reasons for operative mortality compare to how staff describe institutional factors that impact operative mortality outcomes?

**Findings**: In this mixed-methods study, the most frequently perceived reasons for operative mortality in a single-center pediatric heart center over a 4-year period were technical issues and intractable disease, followed by communication. Quantitative findings were comparable to themes emerging from semi-structured interviews, leading to the identification of factors that impact operative mortality outcomes.

**Meaning**: Implementing best medical practices and fostering healthy interpersonal relationships among staff are critical steps to improve operative mortality.

Congenital heart disease (CHD) is the most common birth defect in the United States, impacting 1% of births annually (1). While operative mortality rates for CHD repair, as defined by the Society of Thoracic Surgeons (STS), have improved since the discipline’s inception, recent years show a plateau. One multicenter study reported a 2.6% mortality rate in 2009–2010, with a similar rate of 2.68% reported over a decade later (2, 3). While demographic and physiologic risk factors such as age, weight, and risk adjusted score are well described (4), the influence of other extrinsic factors remains unclear. There is reason to believe that the gaps in the literature, which quantitative studies have failed to fill, may be addressed by mixed-methods research (5).

Qualitative research from noncardiac disciplines have linked better organizational and workplace culture to improved patient outcomes (6), while incivility among coworkers in tertiary hospitals had a negative effect on patient safety and outcomes (7). Other groups have devised implementation plans for influencing more positive organizational cultures (8). Identifying areas for improvement and current areas of success is critical to improving organizational culture and quality of care (9).

Over the past 5 years, Children’s National Heart Center underwent significant changes including construction of a remote command center to monitor all patients from a central location and significant leadership turnover. Following a 365-day period of 0% operative mortality (from June 2023 to June 2024), a mixed-methods study was proposed to identify perceived factors contributing to operative mortality outcomes. The following research questions were answered:

1) What are the descriptive characteristics of patients with CHD who experienced an operative mortality in our single institution from January 2020 to June 2023?
2) What are the most prevalent perceived reasons for operative mortalities seen at our institution?
3) How do staff describe institutional factors that impact operative mortality outcomes?
4) How do the perceived reasons for operative mortality compare to how staff describe institutional factors that impact operative mortality outcomes?

## MATERIALS AND METHODS

### Study Design

A convergent mixed-methods design combined quantitative and qualitative data to identify similarities between reasons for operative mortality and staff’s descriptions of institutional factors influencing these outcomes. Operative mortality was defined according to the Society of Thoracic Surgery as any in-hospital deaths (including those transferred to outside facility) following an index surgical procedure or within 30 days of surgery. The study was approved by the Children’s National Hospital Institutional Review Board on July 30, 2024 (STUDY00001123 titled “Exploring Institutional Factors that Influence Operative Mortality Outcomes in CHD”) and all procedures contributing to this work comply with the Helsinki Declaration of 1975. Consent was waived for the retrospective review of patient records, and verbal consent was obtained for interviews.

### Quantitative Data Collection

A retrospective review of medical records was conducted to describe patient characteristics associated with operative mortality between January 2020 and June 2023, the period immediately preceding a 365-day operative mortality-free period (from June 2023 to June 2024). January 2020 was chosen as the starting point to overlap cardiac surgery leadership changes.

Records were reviewed by a multidisciplinary team including a cardiac surgeon, intensivist, cardiologist, two nurses (including the quality improvement nurse lead), and four research staff members. The team identified clinical characteristics of the patients and categorized perceived influential reasons for operative mortality. For each case, a primary reason was identified, followed by secondary and tertiary reasons, if applicable. Primary reasons were felt to be the most important factor related to the demise of the patients, whereas secondary and tertiary reasons were felt to be attributable, but when taken alone were not perceived to be as influential. Disagreements were discussed among the team until consensus was achieved.

Through team consensus, five major reasons for operative mortality were defined: technical failure, communication issues, intractable disease, management strategy, and decision-making. Definitions and examples of characterizations are found in **Table 1**.

**TABLE 1.** The Five Reasons for the Observed Cases of Operative Mortality, As Defined Through Consensus by the Multidisciplinary Research Team

Reason	Definition	Examples/Scenarios
Technical failure	An inability to perform surgical or clinical skills needed to sustain the patient’s recovery	Missed diagnosis on imaging; errors during a surgical procedure; ineffective implementation of extracorporeal cardiopulmonary resuscitation in a post-surgical setting
Intractable disease	The inability to effectively treat the patient due to underlying medical conditions	Patient very unlikely to recover post-surgery, such as a premature infant with severe mitral stenosis/aortic atresia and pulmonary vein stenosis
Communication	Delays or complete lack of information exchange between and among clinical teams about the patient’s conditions	Lack of timely discussion between teams about the severity of the patient’s condition; lack of any type of discussion to prompt taking action
Management strategy	Ineffective strategies and mechanisms that led to the demise of the patient	Sending a patient to an acute rehabilitation center that was unable to manage such complex patients; utilizing inefficient strategies for extracorporeal membrane oxygenation deployment
Decision-making	The inability to perform an action timely and accurately	Delayed decision to perform additional procedures after an index surgery; deciding to operate when it was known that the condition was futile

### Qualitative Data Collection

Purposive sampling was used to recruit participants. Inclusion criteria included all staff members and administrators from Children’s National Hospital who were employed within the Heart Center since 2020. A total of 55 employee participants were included and recruited for interviews. Of those recruited, four declined, five had scheduling issues, and 23 did not respond after three attempted contacts, leaving 21 interview participants consisting of cardiac surgeons, cardiac intensivists, cardiologists, cardiac anesthesiologists, perfusionists, operative room (OR) nursing team members, and nursing leaders on the cardiac ICU (CICU) and the acute care cardiology unit.

A team of research coordinators conducted semi-structured interviews using a guide developed based on literature from other specialties (6, 9). Interview questions are shown in **eTable 1** (https://links.lww.com/CCX/B648).

### Quantitative Analysis

Characteristics of patients who had an operative mortality were reported using descriptive analysis. Patient characteristics included sex, gestational age, birth weight, age at surgery, diagnosis, chromosomal and syndromic anomalies, type of procedure, STS-European Association for Cardio-Thoracic Surgery (STAT) category, and postoperative length of stay. All patient characteristics and perceived reasons for operative mortality were reported as frequencies and percentages, median and interquartile range, or mean and sd.

### Qualitative Analysis

Interviews were recorded, transcribed verbatim, and immediately anonymized. Transcription was performed using Otter.ai software. Using NVivo Software (Lumivero, Denver, CO, Version 15), a reflexive thematic analysis approach generated emerging codes, categories, and themes (10). Thematic analysis was performed by a team of three research staff members, and a fourth team member was available to independently resolve disagreements. A complete list of codes, categories, and themes are found in **eTable 2** (https://links.lww.com/CCX/B648). A qualitative validation technique known as member checking was used to ensure accuracy of thematic analysis. This technique involves reaching back out to interview participants following analysis to ensure themes are representative of their expressed thoughts (11).

### Integration

Convergence of quantitative and qualitative data was measured using a joint display (12), where the frequency of primary, secondary, and tertiary reasons for operative mortality that were determined through the retrospective analysis were matched to categories and themes that emerged during interviews.

### Post Hoc Analysis

An additional review of operative mortality cases following the 365-day operative mortality-free period (from July 2024 to June 2025) was conducted. Demographic and clinical characteristics and perceived reasons for operative mortality were described and categorized using the same five reasons in Table 1. The reasons in this group were compared with the perceived reasons for operative mortality observed before the 0% operative mortality period. Additional organizational factors were described, such as the overall bed occupancy rates. A cumulative sum graph reported frequency of operative mortality, STAT 5 category, and extracorporeal membrane oxygenation (ECMO) occurrence before during and after the 0% operative mortality period.

## RESULTS

### Patient Characteristics

The multidisciplinary research team reviewed medical charts of 43 patients who experienced operative mortality between January 2020 and June 2023. Descriptive characteristics are summarized in **Table 2**. The sample included 22 neonates (51%), 16 infants (37%), and five children and adults (11%). Of the cohort, 22 were female (51%). Mean birth weight was 2.7 ± 0.8 kg, and mean gestational age was 37 ± 2.7 weeks. Eleven patients (26%) had syndromic diagnoses, and 18 (42%) had chromosomal abnormalities.

**TABLE 2.** Characteristics of Patients Who Had an Operative Mortality Before the 0% Mortality Period (From January 2020 to June 2023)

Patient Characteristics	Total (*n* = 43)
Sex, *n* (%)	
Male	21 (49)
Female	22 (51)
Weight at birth (kg), mean ± sd	2.7 ± 0.8
Gestational age (wk), mean ± sd	37 ± 2.7
Patients with known syndromic diagnosis, *n* (%)	11 (26)
Patients with known chromosomal abnormalities, *n* (%)	18 (42)
Age group at surgery, *n* (%)	
Neonate	22 (51)
Infant	16 (37)
Children and adults	5 (11)
Society of Thoracic Surgeons-European Association for Cardio-Thoracic Surgery level, *n* (%)	
Category 1	5 (12)
Category 2	7 (16)
Category 3	6 (14)
Category 4	7 (16)
Category 5	17 (39)
Indeterminate	1 (2)
Clinical cause of death, *n* (%)	
Cardiac-related causes	14 (33)
Neurologic causes	10 (23)
Respiratory/pulmonary causes	11 (26)
Infection/sepsis-related causes	4 (9)
Indeterminate	4 (9)

STAT category 5 operations were the most common procedures among the operative mortalities (39%). During the study period, including the 365-day operative mortality-free period, progressively more STAT 5 category procedures were performed. Seven patients who were considered eligible candidates for surgery were redirected to compassionate care where care was withdrawn after discussion with the family over the futility related to the case.

### Reasons for Operative Mortality

The most frequent reasons for operative mortality were technical failure (*n* = 19 [22%]) and intractable disease (*n* = 19 [22%]), followed by communication issues (*n* = 18 [21%]), management strategy (*n* = 15 [18%]), and decision-making (*n* = 14 [16%]).

**Figure 1.**
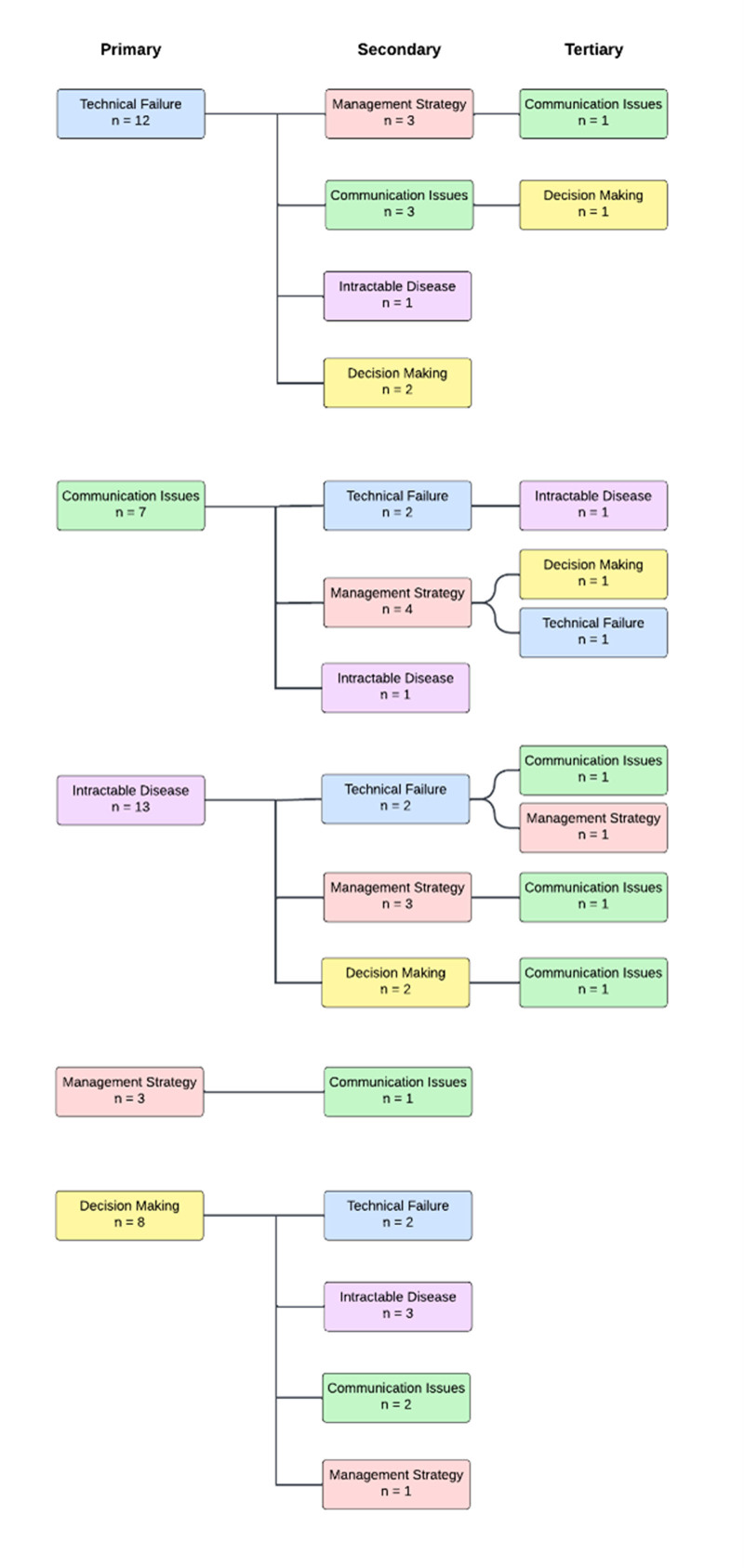
Prevalence of observed primary, secondary, and tertiary reasons for operative mortality outcomes from January 2020 to June 2023.

The most common combination of primary and secondary causes was communication issues leading to management strategy problems, observed in four cases. The only time management strategy occurred as a primary cause with an associated secondary cause, the secondary cause was communication issues, suggesting a potential bidirectional relationship between these factors. This highlights the interplay between communication breakdowns and inadequate management as contributors to mortality outcomes (**Fig. 1**).

### Qualitative Themes

Qualitative analysis generated three themes and 10 categories regarding institutional factors related to operative mortality: (1) Implementing Best Medical Practices, (2) Fostering Healthy Interpersonal Relationships, and (3) Building a Responsive Organizational Structure. The reasons for operative mortality (Technical Failure, Management Strategy, Communication, Decision-Making, and Intractable Disease) were then classified under one of the three themes generated. The frequency of convergence between reasons and themes are summarized in **eTable 3** (https://links.lww.com/CCX/B648).

### Theme 1: Implementing Best Medical Practices

Theme 1, Implementing Best Medical Practices, was concerned with how high-quality clinical practice leads to low operative mortality rates. This theme was comprised of two categories: 1) High Standards of Performance and 2) Strategies to Improve Care. Participants overall emphasized medical acumen, technical skill, and willingness to endure challenging tasks for extended periods of time (e.g., managing delicate patients at bedside for hours at a time) as critically necessary for achieving low mortality rates. Interviewees had high expectations for patient care and stressed the importance of recruiting and retaining talented and hard-working staff that hold these same standards. Developing institutional strategies was also deemed essential for high performance. While the importance of specific strategies in the CICU (e.g., implementing T3 monitoring systems) and OR (e.g., pivoting to a delayed Norwood strategy for single ventricle patients) was covered, broader advocacy for preparedness and the standardization of protocols to minimize the loss of information were predominant.

### Theme 2: Fostering Healthy Interpersonal Relationships

Theme 2 highlighted the need for connection and cohesion among team members within and across disciplines to facilitate optimized patient care. Categories included: 1) Being Open and Able to Change, 2) Interacting with Others, 3) Liking your Job, 4) Incorporating the Voices of Nurses and Advanced Practice Providers, 5) Creating a Psychologically Safe Environment, and 6) Working Together. Participants emphasized how issues with communication were not only detrimental to the culture of the workplace, but that these tense relationships also interfered with patient care, usually due to staff being afraid to speak up about an issue out of fear of retribution. Most often, participants explained that communication between cardiac silos has improved in recent years, and that there has been an overall shift in the style of communication. Staff now listen more to one another and include the diverse perspectives of other specialties and disciplines, which includes increased recognition and respect for opinions and expertise of our nurses. Participants also emphasized that working together encompasses more than just communicating with one another but also involves collaboration between and among teams, which requires a sense of cohesion and a shared mental model. Participants alluded to the idea that while there may not be direct, measurable associations between team satisfaction and morale and patient outcomes, greater cohesion and generally enjoying working with your team leads to positivity, greater collaboration, and a toxicity-free culture, which indirectly improves patient care.

### Theme 3: Building a Responsive Organizational Structure

Theme 3 focused on building a responsive organizational structure to improve operative mortality. This theme consisted of two categories: 1) Having the Resources to Perform and 2) Being an Effective Leader. A responsive structure was described as one that addresses the needs and well-being of staff, ensures adequate resources, and provides support for patients and their families. Staff emphasized the importance of both horizontal support among colleagues and vertical support between them and their leaders and mentees in fostering a responsive environment. They highlighted the need to appropriately use the expertise of medical staff while avoiding unreasonable schedules (e.g., 24-hr shifts) or excessive workloads, which can lead to role strain and negatively impact the work environment and, in turn, patient outcomes. Last, staff participants underlined how leadership styles and training (e.g., formal training or mentorship) significantly influence team morale and cohesion. For example, team activities or events that encourage more one-on-one time with leaders, such as “Lunch with Leaders” or Town Hall meetings, provide a safe space to exchange ideas, express concerns, and communicate team needs to leadership. These interactions help foster mutual understanding, address team demands effectively and drive organizational improvements.

### Post Hoc Findings

A total of 11 patients had an operative mortality from July 2024 to June 2025. Compared with the cohort of patients reviewed in the years preceding the 0% operative mortality period, characteristics were similar in both groups (**Table 3**). The most frequent reason for operative mortality in this time period was intractable disease (*n* = 10 [53%]). Eight of 10 were categorized as primary reasons. Management strategy (*n* = 3 [16%]) and decision-making (*n* = 3 [16%]) were both the next most frequent causes, although they were both only categorized as secondary and tertiary reasons. Technical failure (*n* = 2 [11%]) was cited as the primary perceived reason in two cases and communication (*n* = 1 [5%]) was identified as a tertiary reason in one case.

**TABLE 3.** Characteristics of Patients Who Had an Operative Mortality After the 0% Mortality Period From July 2024 to June 2025

Patient Characteristics	Total (*n* = 11)
Sex, *n* (%)	
Male	6 (55)
Female	5 (45)
Weight at birth (kg), mean ± sd	2.7 ± 1.0
Gestational age (wk), mean ± sd	36 ± 4.4
Patients with known syndromic diagnosis, *n* (%)	5 (45)
Patients with known chromosomal abnormalities, *n* (%)	3 (27)
Age group at surgery, *n* (%)	
Neonate	6 (55)
Infant	5 (45)
Children and adults	0
Society of Thoracic Surgeons-European Association for Cardio-Thoracic Surgery level, *n* (%)	
Category 1	0
Category 2	4 (36)
Category 3	2 (18)
Category 4	1 (9)
Category 5	4 (36)
Clinical cause of death, *n* (%)	
Cardiac-related causes	2 (18)
Neurologic causes	2 (18)
Respiratory/pulmonary causes	3 (27)
Infection/sepsis-related causes	1 (9)
Multiple organ failure	2 (18)
Hemorrhage	1 (9)
^a^Perceived reasons for operative mortality between July 2024 and October 2025, *n* (%)	
Technical failure	2 (11)
Communication issues	1 (5)
Intractable disease	10 (53)
Management strategy	3 (16)
Decision-making	3 (16)

a Some patients were assigned a primary, secondary, and tertiary reason for operative mortality, so total number of reasons is *n* = 19.

**Figure 2.**
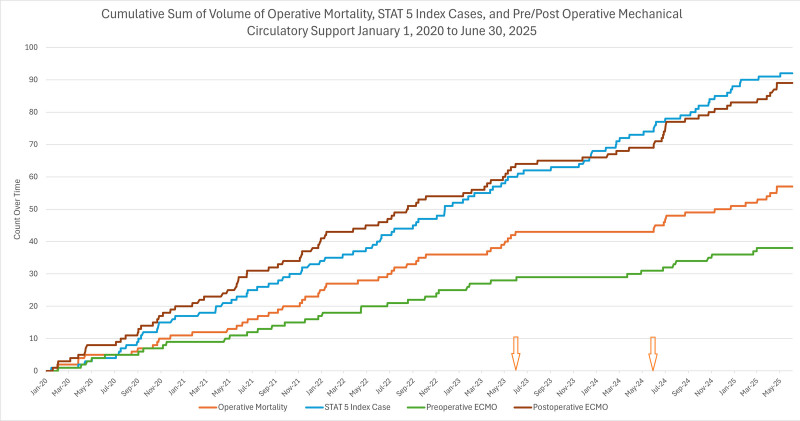
A cumulative sum illustration of the number of operative mortality (*orange*), Society of Thoracic Surgeons-European Association for Cardio-Thoracic Surgery (STAT) category 5 procedures (*blue*), preoperative extracorporeal membrane oxygenation (ECMO; *green*), and postoperative ECMO (*brown*) in relation to the number of cases performed in a single-center institution. The *orange arrows* indicate the start and end of the 0% operative mortality period, which lasted from June 2023 to June 2024.

**Figure 2** shows the cumulative sum of all operative mortalities, preoperative and postoperative ECMO procedures, as well as STAT category 5 procedures performed before, during, and after the 365-day 0% operative mortality period. The number of preoperative and postoperative ECMO procedures decreased in the 0% operative mortality period, as indicated by the straight green and burgundy color lines in Figure 2, while the number of STAT category 5 procedures increased over this same period. During the 0% operative mortality period, the average bed occupancy rate was 18.5 ± 2.0. In the period after, STAT category 5 procedures continued to increase, while there were moderate increases in postoperative ECMO utilization and smaller increases in preoperative ECMO utilizations. The average bed occupancy rate was 22.5 ± 1.3.

## DISCUSSION

In an ever-changing healthcare landscape, leadership values such as communication and team building are valuable, but often ineffectively fostered (13). Assessing teams with successful outcomes can yield insights into which factors are most consequential for safety (14) and can identify values that healthcare leaders should embody. Our Heart Center achieved 0% operative mortality over a 365-day long period, providing an opportunity to codify aspects of a successful pediatric heart center and to understand workplace culture and its impact on care.

Technical failure and intractable disease were the most frequent reasons for operative mortality, converging most often with the qualitative theme of high standards and best practice (eTable 3, https://links.lww.com/CCX/B648). The frequency of these reasons likely reflects the volume of STAT 5 category procedures (Fig. 1). Qualitative data revealed that interacting with others in a timely, effective manner was the most important institutional factor. While communication was cited less frequently, qualitative data showed that timely and effective interactions were essential to improving care, demonstrating a possible direct and indirect link to operative mortality. Our findings are consistent with studies that show organizational structure hinders communication and trickles down to patient outcomes, with preoperative planning and effective handoff techniques being critical for safety (15, 16). This highlights the need to improve early and frequent communication and establish environments that promote communication without fear of retribution.

Our study adds to the literature by identifying environment, culture, and team dynamic factors that impact patient outcomes within a pediatric cardiac center. Our analysis mirrors work done by James Reason (14), who delineates between “active failures” occurring during direct patient contact and the “latent conditions,” which are primarily responsible for these direct failures. The retrospective review of individual operative mortality cases largely focused on these directly responsible elements. The process of matching these reasons for operative mortality to the themes generated through the qualitative interviews reflected a difference between direct factors related to operative mortality and the underlying factors (the latent causes) that are not as readily attributable to deaths.

Data gathered from qualitative interviews exposed latent causes that would otherwise be hidden through chart review. For example, “Being an Effective Leader” was cited infrequently, staff described an overall improvement in leadership engagement and feedback opportunities, indirectly impacting operative mortality through better culture and team satisfaction. The literature shows leaders who promote psychologically safe environments create better patient safety and outcomes (15, 16). We have also facilitated better communication with the establishment of a multidisciplinary case conference in December of 2021, which was intended to shorten the duration between patient contact and decision-making and ensure a shared mental model. This process allows staff to call for a case conference to discuss recent investigations, change in clinical status or discuss big picture plan of care, and takes place in addition to the weekly surgical case conference.

“Liking Your Job” was another category that emerged from interviews but did not converge with quantitative data, including codes related to cohesion, team member familiarity, team satisfaction, and morale. While these categories were not observed in our chart review, they may have an impact on operative mortality by indirectly impacting staff collaboration. Similarly, high turnover rates may decrease staff familiarity with specialized processes. Studies identified that greater familiarity among surgical teams may have indirectly benefited patients by increasing efficiency (17–21).

A post-analysis was conducted after the year of 0% operative mortality to assess which key thematic factors were present during the period when rates began to rise again. Bed occupancy rose, and we had a major shift in staff, resulting in a higher ratio of senior to junior attendings in the CICU and OR. Enthusiasm after a year without operative mortality may have also encouraged the performance of more difficult cases. However, intractable disease was the most frequent reason, suggesting that interpersonal relationships and effective strategies have not necessarily fallen apart. These results highlight the need for more standardized palliative care protocols in cases where intervention has a greater probability of doing more harm than good.

To limit recall bias, a multidisciplinary team assigned reasons for operative mortality through triangulation of medical records, and discussion notes from Critical Event Review meetings—in-depth discussions following an operative mortality. It is also important to note that while we have referred to the retrospective review of data as accounting for the perceived “reasons” for operative mortality, this review was a quality improvement effort, and all analysis was done in hindsight, therefore eliminating the ability to imply causality. Response bias was mitigated by using experienced research coordinators with prior qualitative research backgrounds to recruit employees and maintain complete participant anonymity. Trustworthiness of qualitative analysis was ensured by performing member checking to increase credibility (21). The interview guide and groups of codes, categories and themes were also included for transparency and to allow for transferability to future researchers (20). Finally, interviews were performed during and directly after the period where we experienced 0% operative mortality, so interviewees may have been more positive in their responses.

## CONCLUSIONS

Our study found a greater convergence between observed technical failures and communication with the first and second qualitative themes. As our Heart Center achieved, albeit transiently, a 365-day zero operative mortality period, we identified issues with previous care strategies and found various institutional factors may have played a role in this experience. While operative mortality is inevitable, this study helps examine current practices to potentially improve the care of the most complex patients. Findings from this study illustrate what institutional factors are responsible for ineffective patient care and highlight the importance of developing strategies to enhance communication throughout the patients’ time in the hospital. Future work should implement and test these strategies to measure their impact on patient outcomes.

## ACKNOWLEDGMENTS

We thank the staff participants who volunteered to be interviewed and provided valuable insight into the qualitative portion of this study.

## Supplementary Material
