# Supplementary material for: Using Mixed-Methods Research to Address Stagnant Operative Mortality Rates in Congenital Heart Surgery
Source: Crit Care Explor. 2026 Jul 1;8(7):e1435. doi: 10.1097/CCE.0000000000001435 (PMC13313730; doi:10.1097/CCE.0000000000001435)
Supplement: Supplementary file 1 [file cc9-8-e1435-s001.pdf]

Supplementary Materials

Table of Contents

eTable 1. Copy of the Semi-structured interview questions. 2

eTable 2. Complete copy of all codes, categories and themes that emerged from the qualitative interviews. 3

eTable 3. Joint Display to illustrate the convergence of quantitative and qualitative results. 7

eTable 1. Copy of the Semi-structured interview questions.

**Semi Structured Qualitative Interview Questions**
What factors do you believe influence patient safety following cardiac surgery?
How do you think team environment and work culture influence patient outcomes?
How would you describe your ideal work environment when it comes to working with others to ensure patient care is optimized?
How do you work with your team to ensure quality of care with the most complex patients?
Think about a time when you and your team were faced with a complicated case fraught with many risks that ended up having a positive outcome?
Who was involved in that case? (no names, just roles)
What team strategies were used?
Why was it such a positive outcome?
Overall, what do you believe was the most important drivers of the improved operative mortality outcomes that we experienced in the past year?

eTable 2. Complete copy of all codes, categories and themes that emerged from the qualitative interviews.

Name of Theme, Category, and Code	Number of Interviewees	Number of Times Mentioned
**Theme #1 - Implementing Effective Medical Practices**		
Establishing Quality Care Strategies		
Crisis Management	8	10
Data-Driven Management	2	2
Decision making	2	2
in the moment decision making	4	5
Defining goals	5	5
Being Proactive	2	7
Improving Treatment Strategies	5	7
CICU Patient Management	9	12
Handoff	9	11
HKU Strategy	1	1
HLHS Strategies	3	3
Operative Strategy	11	14
Perfusion Strategy	2	3
Palliative Management	3	4
Parent-Family Resources	2	3
Preparedness	8	12
Selectiveness	1	1
Standardization	11	15
High Standard of Performance		
Attention to detail	3	3
Hard Work	5	5

Color Key
Theme
Category
Code

High Expectation	6	9
**Theme #2 - Fostering Healthy Interpersonal Relationships**		
Being Open and Able to Change		
Adaptation	2	2
Cognitive flexibility	8	10
COVID Disruption	4	5
Open-mindedness	11	13
Reflection	1	1
Room for improvement	10	16
Creating a Psychologically Safe Environment		
Moral Distress	4	5
Psychologically Safe Environment - NO	4	5
Psychologically Safe Environment - YES	9	16
Unafraid to speak up	8	15
Toxicity	3	7
Incorporating the Voices of Nurses and Advanced Practice Providers (APPs)		
Listening to Nurses and APPs	5	6
Nurse and APPs Inclusion	5	9
Nursing and APPs Retention	2	3
Outdated Model of Nursing and APPs	1	2
Interacting with others		
Communication	18	60
Communication Style	14	28
Communication with family	2	2
Early Communication	14	17
Shared Decisions Making with Family	1	1

Listening	5	6
Respect	9	12
Diversity & Identity	2	2
Feeling Heard	4	4
Respecting Other Perspectives	8	14
Liking your job		
Cohesion	9	12
Familiarity	7	10
Physical Space Environment	3	4
Team building activity	2	2
Team Satisfaction and Morale	14	18
Working Together		
Collaboration	10	22
Intra-team Support	4	5
Multidisciplinary	13	24
Utilizing Expertise	14	25
Taking the lead	5	5
Teamwork	13	21
Trust	6	14
Shared Mental Model	13	27
**Theme #3 - Building a Responsive Organizational Structure**		
Being an Effective Leader		
Leadership	2	2
Engagement with Staff	8	11
Strong Leadership	3	4
Supporting Staff	2	2
Mentorship	2	5

Micromanaging	2	2
Having the Resources to Perform		
Bandwidth	1	1
Exhaustion	5	7
24hr Shift	1	2
Burnout	2	3
Role Strain	2	2
Experienced Staff	14	25
Organization	1	1
Resource Availability	3	3
Psychological Support	1	1
Responsibility Overload	6	6
Staffing	12	21
Training	4	8
Informal Learning Environment	3	3

eTable 3. Joint Display to illustrate the convergence of quantitative and qualitative results.

**Theme 1. Implementing Effective Medical Practices**			
**Themes and Categories (N=number of quotes)**	**Qualitative quotes**	**Number of Matched Quantitative Data Categories**	**Interpretation of Integrated Results**
High Standards of Performance (N=17)	"So I think it's sort of that attention to detail, the ability to integrate information, and then, keeping that clinical curiosity, at all times, proving to yourself why is this diagnosis you've chosen, rather than, just defaulting to the first one you thought of?"<br><br>"So I think the ideal work environment would be one that involves people who are smart and well trained, professional and believe that the patient is the focus, not the advancement of their career"	• Primary – 11<br>*Technical Failure=11*<br><br>• Secondary – 6<br>*Technical Failure=4*<br>*Management Strategy=2*<br><br>• Tertiary – 1<br>*Technical Failure=1*	The second highest number of deaths were attributed to errors and technical inadequacy in either surgical technique or medical management. The quality of medicine produced is one of the most impactful factors but also one of the most difficult to change.
Establishing Quality Care Strategies (N=117)	"For example, we did change a surgical technique, for example, kids with single ventricle – we're now doing more delayed [Norwood], or hybrid delay, and that's a reflection [of] many reasons. One of the reasons for that is that we've had very poor outcome with our Norwood, And now	• Primary – 23<br>*Technical Failure = 1*<br>*Intractable Disease = 13*<br>*Management Strategy = 3*<br>*Decision Making = 6*<br><br>• Secondary – 18	The highest number of deaths were attributed to strategic medical decisions. In several cases, policies have changed as a direct result of the mortalities. Death attributed to intractable disease

	we've moved to this delayed Norwood, and the outcome is a lot better"<br><br>"Our perception of being a simple patient is incorrect, and they're a lot more complex. so I think we have to be very standardized in how we evaluate and make decisions, and I think things like surgical conference and [mini-]conferences is the physical actualization of that understanding that we have to approach each patient the same"	*Technical Failure = 2*<br>*Intractable Disease =5*<br><br>*Management Strategy = 7*<br>*Decision Making = 4*<br><br>• Tertiary – 4<br><br>*Intractable Disease = 1*<br><br>*Decision Making = 2*<br>*Management Strategy = 1*	were included due to potential use of palliative care or selectivity as an alternate path.
**Theme 2. Fostering Healthy Interpersonal Relationships**			
**Themes and Categories (N=number of quotes)**	**Qualitative quotes**	**Quantitative Data**	**Integration of Results**
Being Open and Able to Change<br><br>(N=46)	"With every clinical change you might have in your mind –what could be driving that clinical change? But you can't fall in love with your [past] diagnosis, right? So every time you have a high heart rate, you have to go back to all the reasons. Could it be arrhythmia? Could it be infection? Could it be fever? Could it be sedation? If it was sedation last time, it doesn't mean	• Primary – 1<br><br>*Decision Making = 1*<br><br>• Secondary – 0<br><br>• Tertiary – 0	This particular case was categorized as an issue with decision making because it was felt that there was an inappropriate decision to proceed with surgery given the patient's condition and it was matched to this category because

	it's sedation this time? Always keeping that questioning attitude for every clinical change, and then really being persistent in working it up."<br><br>Yeah, sort of being anticipatory and not just reacting,… you're thinking three steps ahead, planning for multiple courses that are all three steps ahead. But in your mind, you're looking at all of them and saying, Okay, do I have all the pieces in place for A, B, C and D contingency? Okay, yes, alright, well, it looks like we're going down the A contingency. Nope, we're going down B. So now let's reorient and get everyone on the same page.		the staff failed to be open to other opportunities for this patient.
Interacting with Others (N=173)	"The new surgical team's style of communication was very different from the style of communication that we had previously had, and so just kind of navigating that has been a challenge. I think that we have gotten better at it. We have learned some of the particular quirks of our current surgeons, and have, I think, developed a better way to communicate with them. I think that also	• Primary – 5<br>*Communication = 5*<br><br>• Secondary – 5<br>*Communication = 5*<br><br>• Tertiary – 3<br>*Communication = 3*	The quantitative theme of communication was matched quite often with this category, specifically when staff did not alert others about an issue in a timely manner.

	helps make things safer, because if you can communicate with the surgeon in a way that he understands what's going on and you are on top of that problem, [and you say] ‘here is the solution, here is what I need you to do’, then they're going to be more willing to hear what you're saying.”<br><br>“I spend much more time listening. I don't talk as much during rounds. I don't say anything until the end. Watching the level of those traits in the other providers of how they communicate – what is their acumen like? Do they know that if this is happening, it's probably because of that, watching their professionalism, watching their positivity, and then synthesizing at the end [saying]. ‘That sounds like a great plan, guys, this is good but remember that we're going to close the chest tomorrow. We really have got to focus less on the sedation and the feeds and more on the diuresis’ – Stuff like that – redirecting the plan in a way that it needs to be after people		

	have spoken – instead of before...”		
Liking your Job (N=46)	“My ideal work environment is one where I feel like I'm going to work with my friends. And the reason I say that is not because we are all here to show up and have fun, although it's a nice thing. We spend most of our waking hours here. Most of our waking hours are not with our families, they are here. But I say it because that creates the environment where you can ask those questions. You can disagree. You can have an open conversation; we have a huge center. We all have people that we gravitate to, people that are actually our friends here versus those who are colleagues, right? And there's no doubt that that flow of information is better”<br><br>“I think that if you have a team that comes to work energized, if you have a team that comes to work where each team member is valued – that person inherently is going to work harder, right? [They're] just going to be more invested in the work and is going to work harder. We	• <u>Primary – 0</u><br><br>• <u>Secondary – 0</u><br><br>• <u>Tertiary – 0</u>	There was no convergence between this category and the quantitative portion, although this category may affect culture and environment which indirectly affect patient outcomes.

	all come to work with the same, I hope we all come to work with the same goal of providing the best care that we can. But how we are created at work, or how we are being valued at work, may affect the quality of that care that we aspire to provide for our patients. So I think that again, going back to that whole culture of safety and culture of teamwork, I think that if we all come to work knowing that are part matters – like if I know what I do matters, what I say matters – then I would be more invested, I would be more likely to speak out, I would more likely to seek information that may affect patient care."		
Incorporating the Voices of Nurses and Advanced practice practitioners (APPs) (N=20)	"The decision was whether or not to give him the chance to extubate again, or whether to put in a tracheostomy. And so the nurse practitioner team really fought hard to create a circumstance that would be favorable for extubation. We looked back at the prior extubation attempts and realized that we hadn't made sure he gained weight. We had just stopped a sedation one hour and tried to extubate in the next hour. So, like,	• Primary – 0<br>• Secondary – 0<br>• Tertiary – 0	There were no instances when not listening to nurses led to an operative mortality outcome, however it was felt that this is an underlying issue that may affect culture and team satisfaction, which may indirectly affect patient outcomes.

	really looking back at what had failed before, and then trying to change those factors for the next attempt. And it took us a lot of convincing, like our medical team wanted to trach this patient, and the NP team was like, No, we can do this. And so it took a lot of patience, but also trust of them in us.... And so we extubated him, and he didn't end up leaving the hospital with a tracheostomy. So I think part of that was that it was a conversation between peers. So even though they are the attending physician, and we are the nurse practitioners, it was still like we were viewed as equivalents in terms of, we're all clinicians together."<br><br>"I also think listening to the nurses, was huge. I think we were trying to function as if we had a full staff for a while, even though we had so many floats – and we still – do so many travelers, and not listening to like, "this is dangerous", until there actually was a death, and that ended up having people peel back how many kids we allowed in the Unit until we were like		

	in a safer place. And I think that that was huge and very important."		
Creating a Psychologically Safe Environment (N=48)	"My ideal environment would be not being afraid that I was going to get in trouble for messing up, having, you know, other people be accountable for their accidents as well, on an equal level that you know, like if you make a mistake, it really is the culture of right now, of, if you make a mistake, you're afraid to say something, because you know, are afraid that you're going to get in trouble when it be, that it should be, hey, I made a mistake, and I'm going to tell everybody, because I don't want them to make the same mistake."<br><br>"When people don't feel heard about their concerns or about trajectories of patients, it's hard to speak up again if you've always been shut down every time you bring up a concern."	• Primary – 0<br><br>• Secondary – 0<br><br>• Tertiary – 0	There was no convergence between the reasons for operative mortality and this category. This category was related specifically to instances where participants did not feel that they were in a psychologically safe environment. This may have hindered communication and opportunities to engage in shared decision making, therefore impacting the patient outcomes indirectly.
Working Together (N=116)	"And an environment, where the work is collaborative, where I recognize the surgeon's expertise and appreciate the surgeon's expertise and	• Primary – 3<br>*Communication = 2*<br>*Decision Making = 1*	There were seven instances where multidisciplinary teams failed to work together to communicate

	the work that they put in for the patients. And then vice versa, where the surgeons or the nurses recognize my expertise at the bedside and values that and take that into consideration when we make decisions together for patients. But really a culture of where everybody values one another, well supported so that we're not too overstretched, so that we can take the time to care for our patients.”<br><br>“And the culture is the main manager of what kind of team you can have in this culture. And if the culture in my team is going to affect how much I as a person, as a woman, for example, go and integrate into this team, how much we can trust each other. That culture is going to affect the relationship in a professional level – it will affect the trust that that we can create in this environment. It's not about personal trust. I'm talking about your skills or the clinical skills, [and] how much we can teach each other. Because it's an ongoing process. It's not just ‘you came here at this level and you are going to stay here.’ The goal is	• Secondary – 3<br>*Communication = 2*<br>*Management Strategy = 1*<br><br>• Tertiary – 1<br>*Communication = 1*	when there was a change in care plans and failed to consult certain teams when necessary. Thus, cases with both communication and decision-making were matched to this category.

	[more like] "I will improve you as much as myself [and] we will pull each other up every time.' And then this actually comes with the culture and then trust in this professional level."		
**Themes 3. Building a Responsive Organizational Structure**			
**Themes and Categories**	**Qualitative quotes**	**Quantitative Data**	**Integration of Results**
Having the Resources to Perform<br><br>(N=83)	"I think that the perfect environment to work in is one where we're well supported so that we can provide the expert care that we're all capable to do."<br><br>"Big factor [in achieving a year with no mortality] is staffing. I think that having the appropriate [number] of frontline providers, whether that be like advanced practice providers or fellows, as well as the appropriate number of attendings to be able to manage bigger operations at night, is really important."	• <u>Primary – 0</u><br><br>• <u>Secondary – 1</u><br><br>*Management Strategy = 1*<br><br>• <u>Tertiary – 0</u>	There was only one instance where the observed quantitative results matched the staff's views about which factors were important for maintaining better patient outcomes. In this instance, management strategy was matched to this category because the staff did not have the proper training and therefore were not aware of particular protocol to follow.
Being an Effective Leader	"Making sure that leadership has a pulse on the culture. I think the Town Hall that happened	• <u>Primary – 0</u>	While the reasons for operative mortality did not correspond to the

(N=19)	in 2022 was really good, because I think it was a very eye-opening experience for the leadership. I think sometimes the leadership doesn't know on the ground how people are actually feeling, and then for them to find out that, like we feel micromanaged, we feel XYZ, and those are things that can be fixed I think really helped with the culture and helped with outcomes."<br><br>"There was a culture driver. Leadership, based on feedback from the Heart Center as a whole, supported and promoted a culture change. Just stressing the importance of collaboration, as opposed to in-fighting. And that, I think that proved to be very effective."	• Secondary – 0<br><br>• Tertiary – 0	leadership theme, it was noted that issues with leadership were more of an underlying cause of a negative culture and environment, which were thought to indirectly influence patient outcomes.
